# Editorial: Alternative and complementary methods for the control of infectious diseases in animals

**DOI:** 10.3389/fvets.2022.1015253

**Published:** 2022-09-15

**Authors:** Adeyemi O. Aremu, José Alberto Rosado-Aguilar, Lyndy J. McGaw

**Affiliations:** ^1^School of Life Sciences, College of Agriculture, Engineering and Science, University of KwaZulu-Natal, Durban, South Africa; ^2^Indigenous Knowledge Systems Centre, Faculty of Natural and Agricultural Sciences, North-West University, Mmabatho, South Africa; ^3^Campus of Biological and Agricultural Sciences, Faculty of Veterinary Medicine and Zootechnics, Autonomous University of Yucatan, Mérida, Mexico; ^4^Departamento de Salud Animal, Facultad de Medicina Veterinaria y Zootecnia, Universidad Autónoma de Yucatán, Mérida, Mexico; ^5^Phytomedicine Programme, Department of Paraclinical Sciences, Faculty of Veterinary Science, University of Pretoria, Pretoria, South Africa

**Keywords:** ethnoveterinary, phytomedicine, antimicrobial, anthelmintic, animals

Animal production contributes significantly to the food security, income and wellbeing of humans. However, infectious diseases in production, companion and wild animals often cause serious losses to the economy, in addition to exacerbating food insecurity (1). Conventionally, livestock farmers, animal health clinics and zoos often rely on the extensive use of synthetic chemicals, including antibiotics, anthelmintics and feed supplements to improve meat production and animal welfare (2). Long-term use of chemical drugs has led to serious problems, such as the rapid development of drug-resistant infectious agents and negative consequences for human and animal health (3, 4). As a result, there is a paradigm shift toward the search for alternative or complementary options and more “natural” ways of managing animal health and production (5–7). Alternative methods of animal husbandry and medication are increasingly being recognized for their potential usefulness in contributing to animal welfare, particularly at the level of primary animal health care. It remains pertinent that researchers continuously explore treatments that involve the application of biological control, vaccines, management practices, plants and natural products (1).

Against this background, the current Research Topic brings together different academic disciplines to offer new knowledge regarding alternative and complementary methods useful against infectious diseases in animals. We present a collection of 10 articles, including original research (70%), reviews (20%) and perspectives (10%).

In the face of the multifarious dynamic nature of animal production in Europe, which is partly attributed to political and economic factors, research by Mattalia et al. compared current and past ethnoveterinary practices, and identified trajectories in ethnoveterinary knowledge in northern and southern Eastern Europe. The results identified some patterns common to several countries and to veterinary medicine promoted during the time of the Soviet Union. Using the Netherlands as a case study, Groot et al. provided an insightful perspective on how to further reduce the application of veterinary antibiotics to safe-guard public health. Recommendations include the potential of plant-based products for farm health plans and the importance of monitoring antibiotic use to identify non-registered applications and to set benchmarks. In addition, the need to develop new analytical strategies to monitor the use of antibiotics on farms was emphasized.

Foot-and-mouth disease is recognized as a dangerous infectious disease in livestock. The study by Kass et al. focused on how Estonia addressed the two outbreaks of foot-and-mouth disease in 1952 and 1982. Based on diverse engagements, including written and archival sources as well as interviews with 29 experts, the study explored interesting historical methods used to manage foot-and-mouth disease as well as the practicability of such methods in today's world. Moreover, the importance of plant-based remedies for managing cattle diseases in South Africa was the focus of the review by Chakale et al. An inventory of 310 plants that are known as herbal medicine against 10 categories of cattle diseases in South Africa was provided. The authors revealed that only 21% of these plants have been screened for biological activities (mainly antibacterial effects) and putative safety. The identified gaps in the review open new opportunities for research necessitating urgent attention to promote and explore the benefits of plants in ethnoveterinary medicine.

There is a need to discover new approaches to combat the proliferation of multiple antibiotic resistant pathogens in the dairy sector. The increasing incidence of such pathogens poses a great threat to food sustainability and security, and the health of the public. Bovine mastitis is a major health problem in the dairy industry that results in significant economic losses. The review by Ajose et al. entailed a critical appraisal on how ethnoveterinary medicine can be explored as a viable alternative for combating bovine mastitis in the face of increasing global antimicrobial resistance. The predominant pathogens implicated in causing bovine mastitis include *Streptococcus agalactiae, Staphylococcus aureus, Streptococcus dysgalactiae* and coliform bacteria. The review addressed diverse aspects relating to bovine mastitis in terms of the risk factors, pathogenesis, management, the molecular identification of causative agents, as well as the potential of ethnoveterinary medicine as an alternative therapy. Furthermore, research by Jiang et al. focused on determining rational regimens of cefquinome for the treatment of bovine mastitis caused by *Staphylococcus aureus*. This entailed extensive *ex vitro* pharmacokinetic and pharmacodynamic investigations of cefquinome, which was administered as three consecutive intramammary doses at 75 mg/gland. The authors established that the CO_PD_ for cefquinome against *S*. *aureus* was 2 μg/ml at the recommended dose of 55 mg/gland/12 h. The findings could assist in the treatment of clinical mastitis and reduce the prevalence of drug-resistance, thereby enhancing animal welfare and human health.

The use of plant-based remedies for the treatment of poultry diseases caused by various pathogens remains popular. Olawuwo et al. screened six selected medicinal plants against planktonic and biofilm forms of poultry pathogens (bacterial and fungal strains). In addition, the cellular safety of the plant extracts was assessed. Based on the potential *in vitro* antimicrobial effect and safety, the authors identified *Morinda lucida* extract as a promising candidate for development of an alternative feed additive or supplement in poultry production. In a similar vein, Qinghao Powder (QHP) is a herbal medicine made from the Chinese medicinal plant *Artemisia annua*. In an effort to explore the potential of this plant-based remedy, Wang, Guo et al. investigated the efficacy and safe dose of the petroleum ether (PE) extract of QHP on broiler chickens with coccidiosis induced using *Eimeria tenella* oocysts (Guangdong strain). The authors established that QHP at 0.30 g/kg would be an effective and safe therapy for intermittent treatment of *E*. *tenella*-infected chicks. In another study, Jambwa et al. evaluated the potential development of a poultry phytogenic feed additive from *Senna singueana*. Bioassay-guided fractionation led to the isolation and identification of luteolin. The authors investigated the antibacterial, anti-lipoxygenase and antioxidant activity, as well as the *in vitro* safety of fractions and isolated compounds. Based on the promising bioactivity and low cytotoxicity, *S*. *singueana* was recommended as a promising candidate for the development of poultry phytogenic feed additives.

The residue effect of commonly used antibiotics in the environment, including water bodies, remains a major concern globally (4). Polymyxins including colistin (polymyxin E) are last-resort antibiotics for treatment of multidrug-resistant Gram-negative bacterial infections (Wang, Lv et al.). However, the large quantity of colistin released *via* animal feces into the environment has resulted in increasing incidence of the novel *mcr* mobile colistin resistant gene. As a means of combating this problem, research by Wang et al. revealed that ferrate (VI) oxidation is a highly effective and environment-friendly strategy to degrade colistin in water bodies.

In summary, this Research Topic highlights exciting new research dealing with a range of alternative and complementary methods for the control of infectious diseases in animals. From dairy cows to poultry, several studies provide evidence that there is some light at the end of the tunnel of antibiotic resistance. Reports emphasize that a wide range of medicinal plants are used traditionally to combat various diseases in livestock and this is a largely untapped resource for further investigation. The rising threat of diseases caused by drug resistant pathogens lends further support to the urgent investigation of other means for controlling such pathogens without relying on the development of new antibiotic molecules against which resistance is certain to emerge within a short period.

An approach encompassing various complementary methods for combating infectious animal diseases will certainly assist in improved production animal farming, leading to better food security and animal welfare as well as lowering the risk of spreading zoonoses. Companion animals also stand to benefit from a more holistic approach to animal health, in this way promoting human wellbeing as an added advantage. It is hoped that this Research Topic motivates further investigations in this critical area of exploration, leading to scientific validation of a host of potential alternative and complementary approaches to controlling animal infectious diseases. This will contribute to a multitude of areas involving animal and human health and wellbeing.

## Author contributions

All authors listed have made a substantial, direct, and intellectual contribution to the work and approved it for publication.

## Conflict of interest

The authors declare that the research was conducted in the absence of any commercial or financial relationships that could be construed as a potential conflict of interest.

## Publisher's note

All claims expressed in this article are solely those of the authors and do not necessarily represent those of their affiliated organizations, or those of the publisher, the editors and the reviewers. Any product that may be evaluated in this article, or claim that may be made by its manufacturer, is not guaranteed or endorsed by the publisher.
